# A Case Report of Disseminated Canine Histiocytic Sarcoma in Trinidad and Tobago

**DOI:** 10.3390/vetsci5010009

**Published:** 2018-01-19

**Authors:** Stacy Rajh, Karelma Frontera Acevedo, Gillian Williams, Indira Pargass, Alissa Bally, Rod Suepaul

**Affiliations:** The School of Veterinary Medicine, Faculty of Medical Sciences, University of the West Indies, Trinidad and Tobago; stacyrajh@gmail.com (S.R.); Gillian.Williams@sta.uwi.edu (G.W.); indira.pargass@sta.uwi.edu (I.P.); Alissa.Bally@sta.uwi.edu (A.B.); rsuepaul@sta.uwi.edu (R.S.)

**Keywords:** ocular tumor, canine disseminated histiocytic sarcoma, immunohistochemistry

## Abstract

Ocular histiocytic sarcomas (as a presenting part of disseminated histiocytic sarcoma) are not commonly diagnosed. A 10-year-old female intact Rottweiler presented to the School of Veterinary Medicine, Trinidad with buphthalmia and pain in the left eye. The cornea of the left eye appeared diffusely opaque with a conjunctival mucopurulent ocular discharge. A thorough ophthalmic assessment identified an intraocular proliferative tumor to which a unilateral enucleation was performed, however the animal died soon after. Post mortem examination and light microscopy revealed that the intraocular lesion with visceral macro-metastases was in fact a histiocytic sarcoma. Further to this, immune-phenotyping was performed to confirm the diagnosis of disseminated histiocytic sarcoma. This is the first time such a tumor has been diagnosed in Trinidad and Tobago.

## 1. Introduction

Canine histiocytic disease is the umbrella under which a wide spectrum of proliferative disorders have been characterized according to varying biologic characteristics. This group of diseases include distinct subdivisions; canine cutaneous histiocytomas, canine reactive histiocytosis (cutaneous and systemic forms), and the histiocytic sarcoma complex (localized and disseminated histiocytic sarcoma) [1]. Historically, malignant histiocytic sarcoma has best been recognized in the Bernese mountain dog, in which a familial association was made apparent, however the disease has since been described in other breeds. According to Affolter and Moore [2], of the 39 dogs of varying breeds and gender that were examined, Rottweilers, Bernese mountain dogs, and Golden Retrievers were highly represented. The age group examined extended from 2 to 13 year olds with a female to male ratio of 1.2 to 1. Rottweilers and Golden Retrievers were also prevalent in a study done on ocular canine histiocytic sarcoma [3].

The disseminated neoplastic process of histiocytic sarcomas is an aggressive multisystem disease with the formation of multiple tumors in various organ systems carrying an unfavorable prognosis. Predilection sites for primary lesions include the spleen, lymph node, lung, bone marrow, central nervous system, skin and subcutis, and periarticular and articular tissues of the limbs. Secondary sites are also varied, but typically include the liver and lungs [4]. When histiocytic sarcomas occur in the eye, they tend to be considered part of the disseminated form [3]. These tumors may bear similar histo-morphology to other neoplasms, thus in such an instance, immunohistochemistry is the definitive diagnostic tool for differentiation [5,6].

Histiocytic sarcomas most characteristically express the surface molecule CD18 [6]. However, since this is also present in lymphomas, it is important to exclude lymphoid origin (CD3 and CD79) [1]. They can also be positive for MHC II [6]. For ocular cases, it is recommended to do CD18, and they should be negative for Melan-A [3].

This report describes a case of canine disseminated histiocytic sarcoma based on its morphology and immune-phenotype which is the first report of its type in Trinidad.

## 2. Materials and Methods

A 10-year-old female intact Rottweiler presented to the Veterinary Teaching Hospital (VTH), School of Veterinary Medicine (SVM), The University of the West Indies, St. Augustine (UWI). It was diagnosed with an intra-ocular neoplasm of the left eye, which was subsequently enucleated, fixed in 10% formalin for 48 h, and sent to the veterinary anatomical pathology unit of the SVM for histological examination. The animal subsequently died and the carcass was also submitted to the veterinary anatomical pathology unit of the SVM, where a routine post mortem evaluation was conducted. The grossly visible lesions were identified, and described as needed. The fresh tissues were sectioned and immediately placed in 10% neutral buffered formalin for fixation. All histological tissues were routinely processed, cut with a microtome at 4 µm, and stained with hematoxylin and eosin (H&E). Immunohistochemistry (IHC) was performed using anti-CD18 (Dr. Peter Moore, Davis, CA, USA), anti-MHCII (VMRD, Pullman, WA, USA), and anti-CD3 antibodies (Dako, Carpinteria, CA, USA), at the Pathology Diagnostic Laboratory, College of Veterinary Medicine, University of Georgia. Basic steps include deparaffinization and antigen retrieval, endogenous peroxidase blocking, and power block. Then the slides were incubated with primary antibody at the following dilutions: CD3—1:1000, CD18—1:50, and MHCII—1:5000. They were then incubated with appropriate secondary antibodies, which were biotinylated anti-rabbit (Vector Lab, Burlingame, CA, USA) for CD3 and biotinylated anti-mouse (Vector Lab, Burlingame, CA, USA). Then they were incubated with streptavidin and horseradish peroxidase, followed by chromogen development using 3,3′-diaminobenzidine (DAB) and hematoxylin counterstaining. All of these used canine lymph nodes for positive control and isotype control serum for negative control.

## 3. Results

### 3.1. Signalment, History, and Clinical Assessment

A 10-year-old female intact Rottweiler, with a satisfactory body conditioning presented to the VTH-UWI for a clinical evaluation. The patient had buphthalmia and pain in the left eye one-week prior to consultation. The left eye was diffusely opaque, exuding a mucopurulent discharge. An ophthalmic and ultra-sonographic evaluation was conducted immediately. The results indicated that the right eye was clinically normal. Conversely, the left eye displayed diffuse corneal edema, conjunctival discharge, and an increased intraocular pressure (39 mmHg) with a lack of fundic detail. A diagnosis of a unilateral intraocular tumor and secondary glaucoma of the left eye was assigned.

Consequently, ultra-sonographic findings displayed evidence of retinal detachment. The focally extensive ocular mass was assessed within the posterior chamber, measuring 13 mm at the temporal aspect of the globe. Medical management involved the use of Lantanoprost^®^ eye drops. However, a unilateral enucleation was the recommended course of therapy given the extent of the lesion. Subsequently, the left eye was surgically removed with the concomitant excision of a cutaneous tumor on 6 November 2014. Bloodwork indicated only an increased level in serum creatinine, but the animal was eating and bright, alert, and responsive prior to surgery. She became lethargic and passed dark tarry stool (melena) on 7 November 2014, and died the day after (two days after surgery).

### 3.2. Gross Examination

**Figure 1:**
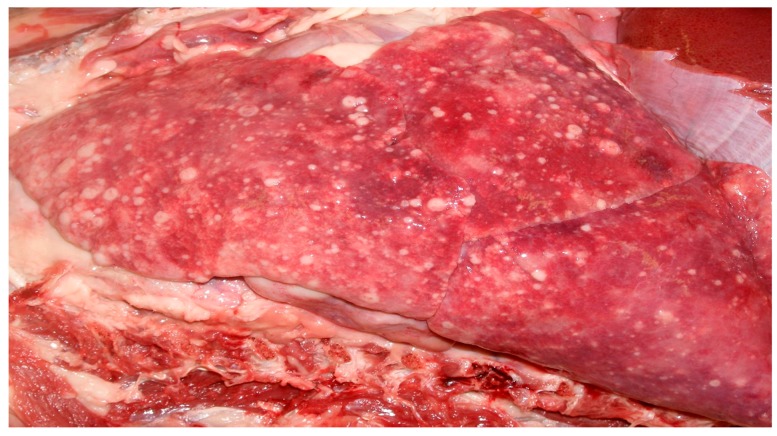
The lungs, in situ, with multifocal pale yellow to white masses.

This dog was given a body condition score of 5/5 due to excessive discernible fat stores. Throughout the pulmonary parenchyma, there were multifocal white to pale yellow, semi soft masses ranging from 100 × 120 mm to 10 × 20 mm, with the largest occupying over 50% of the right cranial lobe (Figure 1). Additionally, multiple masses were present in the liver (10 × 20 mm to 50 × 60 mm) and bulging out of the kidney surface (40 × 50 mm to 20 × 30 mm) resulting in hepatomegaly and bilateral renomegaly. The sub-mandibular and prescapular lymph nodes were enlarged and measured (50 × 30 mm) and (60 × 40 mm) respectively, due to macro-metastases. The intestinal lumen contained bloody soft contents progressing to melena. Differentiation of the primary tumor site was not feasible grossly.

### 3.3. Histopathological Examination

**Figure 2:**
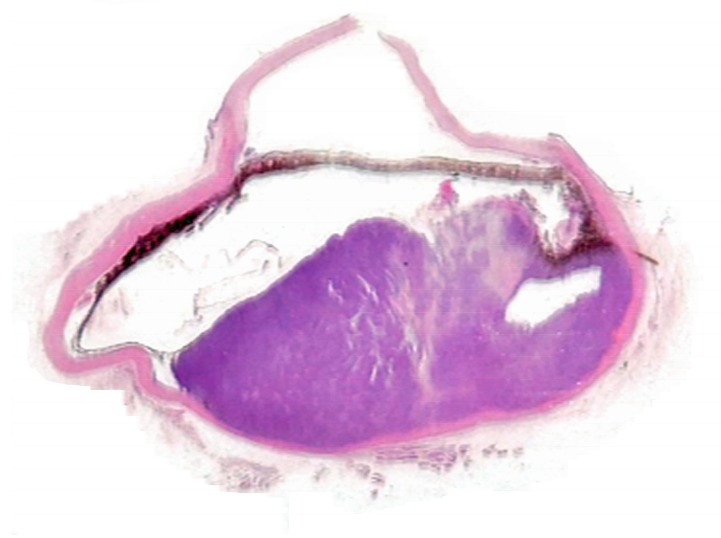
The left eye, where a large mass occupied approximately 70% of the posterior chamber.

**Figure 3:**
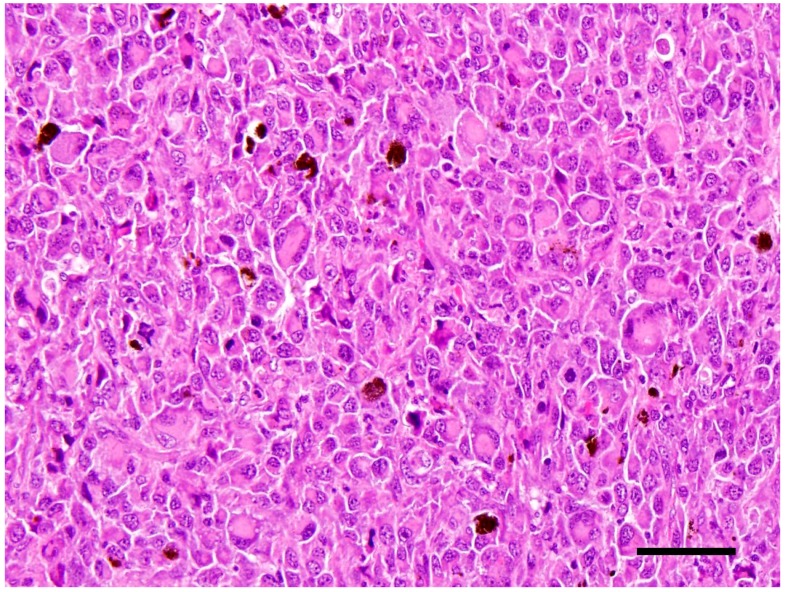
Neoplastic round cells in the eye. They displayed marked anisocytosis and anisokaryosis with moderate to abundant amounts of eosinophilic cytoplasm. Nuclei were singular to multiple and reniform to rounded nuclei with heterochromatin; H&E 40×; bar = 50 μm.

In the left eye, over 60% of the retina was effaced by a large mass that occupied approximately 70% of the posterior chamber (Figure 2). The mass is composed of densely packed neoplastic round cells which displayed marked anisocytosis and anisokaryosis with moderate to abundant amounts of eosinophilic cytoplasm. Nuclei were singular to multiple and reniform to rounded nuclei with heterochromatin (Figure 3). Mitoses ranged from 2–4 per 10 high power (40×) field. There was also a pre-iridial fibrillary membrane. The adjacent retina was detached with hypertrophy of the pigmentary epithelium and loss of most of the remaining layers, leaving only remnants of the inner nuclear layer. The irido-corneal angles were bilaterally occluded. The cornea was thin and bulging with multiple peripheral infiltrates of small numbers of melanocytes. The lens could not be assessed in the section because it had been removed prior to sectioning. Multiple tissues inclusive of focal areas in the pulmonary, renal and hepatic parenchyma, and the peripheral lymph nodes contained neoplastic cells with similar histological characteristics to those in the eye. These findings corroborated with a diagnosis of disseminated histiocytic sarcoma. The right eye was not taken for histopathological examination.

### 3.4. Immunohistochemistry

**Figure 4:**
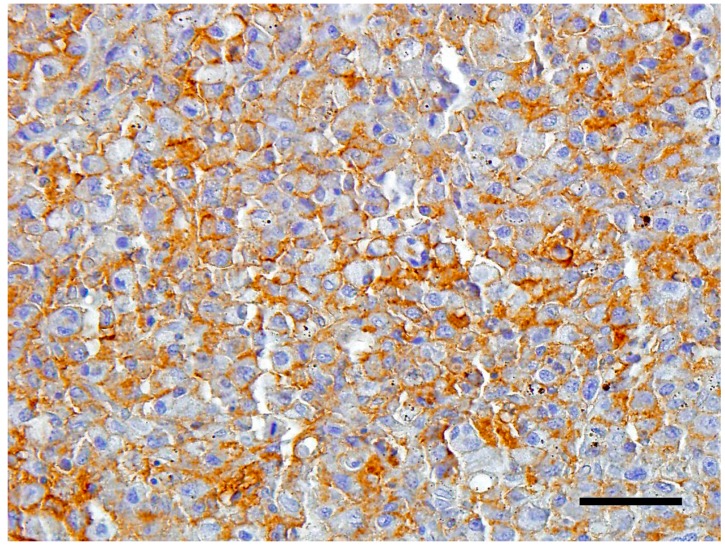
Photomicrographs of CD18 immunocytochemistry of tumor cells. 3,3′-diaminobenzidine chromogen with hematoxylin counterstain; bar = 50 µm.

The neoplastic cells expressed strong plasmalemmal staining for CD18 (Figure 4). The cells were weakly positive for MHC class II, and negative for CD3.

## 4. Discussion

This case presented with the initial complaint of an intraocular lesion manifesting itself as clinical buphthalmia. Histiocytic sarcoma has reportedly been an uncommon primary intraocular neoplasm [1], although recent work has suggested that it should be included in the differentials for intraocular masses, particularly in breeds where histiocytic sarcoma is prevalent [3]. Clinical signs typically are vague and non-specific but will vary according to the organ of involvement [4]. As observed with other cases [3], the initial intraocular mass was the initial presentation of the disseminated tumor. Upon further evaluation, as a result of the widespread metastases at the time of tumor recognition and diagnosis, it was difficult to determine the primary site of origin. Therefore, a diagnosis of disseminated histiocytic sarcoma was assigned as a result of its spread in several organ systems beyond the sites of local lymph nodes. Additionally, metastases to the kidney and destructive mass formation would have caused elevated serum creatinine levels secondary to kidney disease.

Synonymous with the human counterpart, the accurate diagnosis of canine localized and disseminated histiocytic sarcomas in dogs requires immunophenotypic evaluation for confirmation [1]. Furthermore, because the other common intraocular tumor of dogs is ocular melanoma, it is important to separate both types to provide accurate (and adequate) prognosis [3]. Canine ocular melanoma carries a benign prognosis with regards to metastases, while histiocytic sarcoma is an aggressive tumor with a shorter survival time. In fact, in the study by Naranjo [3], although the dogs initially presented with ocular clinical signs, the majority of them eventually died of complications resulting from the disseminated histiocytic sarcoma, just like in this case. This is the first report of such a case of disseminated ocular histiocytic sarcoma in Trinidad.
